# Primary leiomyosarcoma originating from the deep femoral vein initially presenting as chronic deep vein thrombosis: A case report and review of literature

**DOI:** 10.1097/MD.0000000000048155

**Published:** 2026-04-10

**Authors:** Eunju Jang, Ki Yoon Moon, Sun Cheol Park, Jang Yong Kim, Sang Seob Yun

**Affiliations:** a Division of Vascular and Transplant Surgery, Department of Surgery, Seoul St. Mary’s Hospital, College of Medicine, The Catholic University of Korea, Seoul, Republic of Korea.

**Keywords:** bypass, femoral vein, leiomyosarcoma, vascular surgery

## Abstract

Leiomyosarcoma (LMS) is a rare and aggressive smooth muscle tumor, and venous leiomyosarcoma (VLMS) is an uncommon subtype, with majority of the cases involving the vena cava. We report a case of a 56-year-old patient initially diagnosed as deep vein thrombosis (DVT) in the common femoral vein who was later diagnosed with primary VLMS originating from the deep femoral vein. Despite anticoagulation therapy, leg edema persisted and imaging studies revealed an intraluminal mass with increased extent and size. Initial duplex ultrasound suggested chronic DVT; however subsequent contrast-enhanced computed tomography and magnetic resonance imaging performed during follow-up demonstrated interval growth of an intraluminal mass, and positron emission tomography-computed tomography later confirmed metabolically active disease, leading to the diagnosis of VLMS. Surgical resection of the mass was performed, followed by reconstruction of the femoral vein using an expanded polytetrafluoroethylene graft. Histopathologic examination confirmed the diagnosis of LMS. The patient had an uneventful postoperative recovery and was referred for adjuvant therapy, including chemotherapy and radiotherapy. This case highlights the importance of a multimodal diagnostic approach in differentiating neoplasms from blood clots and underscores the need for a high level of suspicion when DVT presents with atypical features. Early diagnosis and wide surgical excision with the aim of achieving negative margins are essential for successful management of VLMS.

## 1. Introduction

Leiomyosarcoma (LMS) is a rare malignant tumor that originates from smooth muscle connective tissue, accounting for approximately 5% to 10% of all soft tissue sarcomas.^[1]^ LMS can develop in any area of the body containing smooth muscle cells. In clinical practice, primary vascular LMS represents a diagnostic challenge, as it can often be misdiagnosed as deep venous thrombosis (DVT) leading to delayed diagnosis and treatment.^[2,3]^

We report a case of a patient initially diagnosed as DVT in the common femoral vein. On closer inspection using a multimodal diagnostic approach, the patient was later diagnosed with primary venous LMS originating from the deep femoral vein.

## 2. Case report

**Figure 1.**
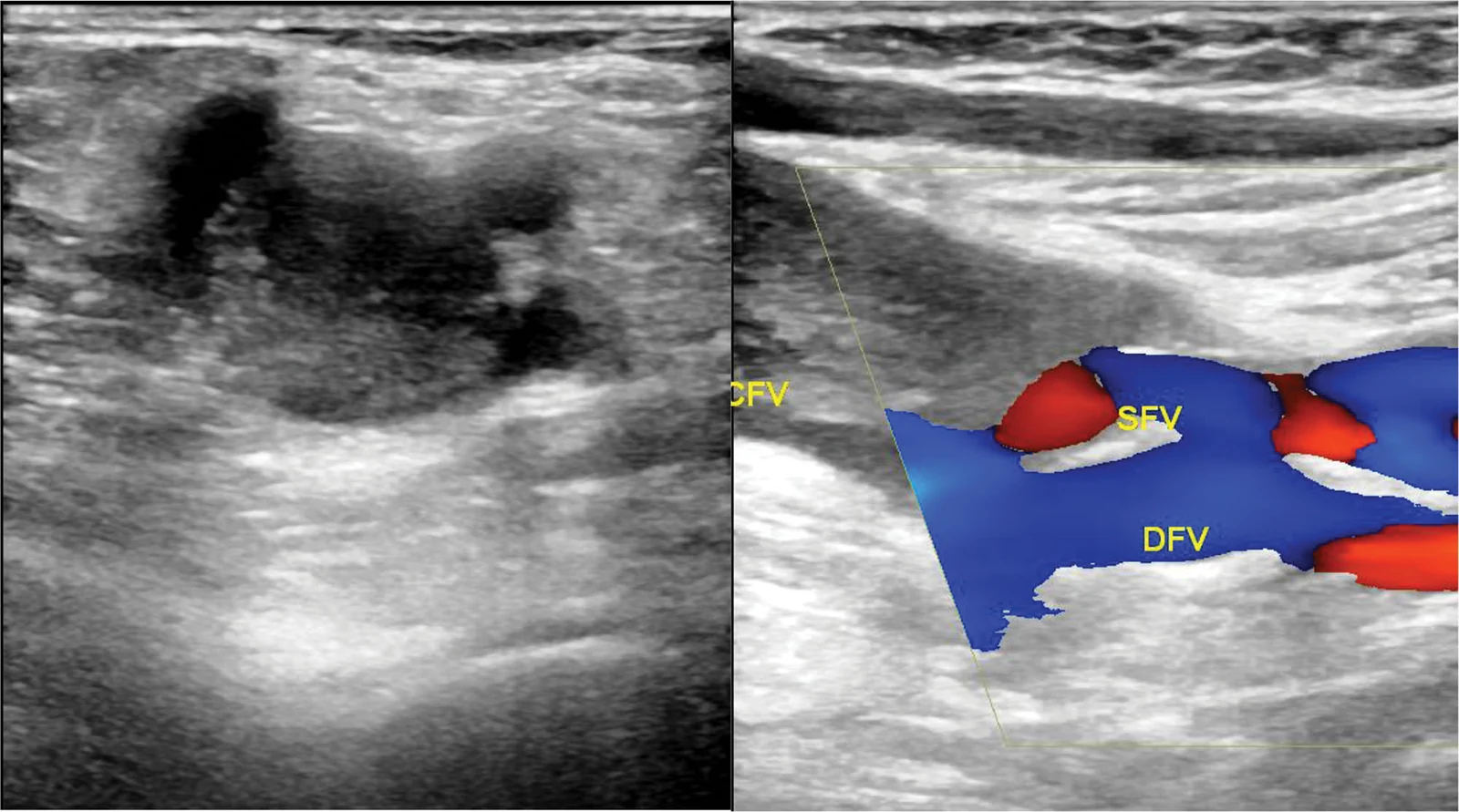
Duplex ultrasound of the left femoral vein showing an intraluminal hypoechoic lesion within the femoral vein at initial examination. CFV = common femoral vein, DFV = deep femoral vein, SFV = superficial femoral vein.

In July 2024, a 56-year-old Asian woman visited our outpatient clinic reporting mild swelling in her left leg which had persisted for 1 month. She was a homemaker with no prior medical issues or surgical interventions, and laboratory results, including D-dimer levels, were normal. Prior to her referral, a duplex ultrasound examination performed at a local clinic revealed an intraluminal hypoechoic lesion involving the left common femoral vein (Fig. 1). Based on the presence of a hypoechoic intraluminal lesion and persistent localized edema without systemic symptoms, the patient was initially diagnosed with chronic DVT and was started on direct oral anticoagulant (DOAC) therapy which was continued during the diagnostic period. Routine follow-up assessments were done at 3 month intervals.

Despite good compliance with anticoagulation therapy, the patient’s symptoms did not resolve. A follow-up duplex examination confirmed that the hypoechoic mass involving the femoral vein remained unchanged. The initial duplex examination had shown a 2 cm lesion localized at the common femoral vein occupying the entire lumen, while the deep femoral vein and superficial femoral vein were patent. Follow-up examination after 3 months showed no significant change. However, at 1 year follow-up, duplex ultrasound indicated a slight increase in the diameter of the lesion to 2.7 cm, with extension into the deep femoral vein.

**Figure 2.**
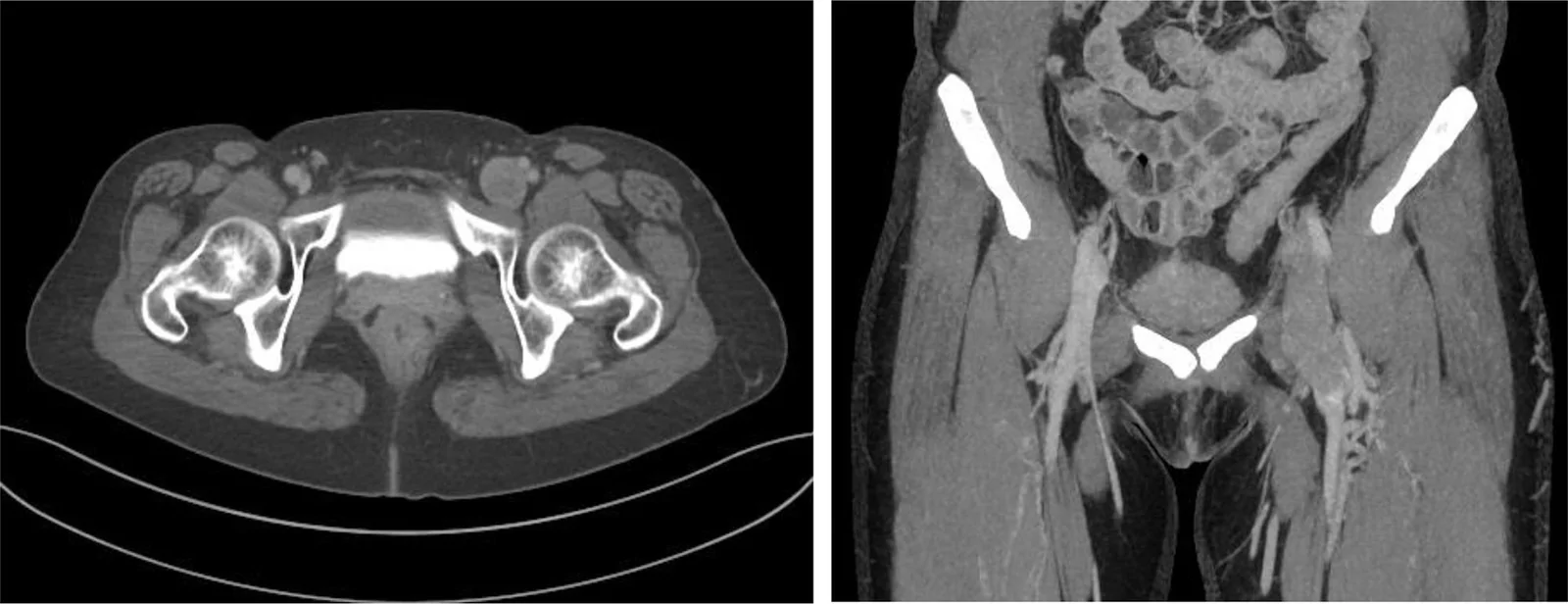
Axial and coronal image of the contrast-enhanced computed tomography (CT) result after 1 year of anticoagulation. CT = computed tomography.

Due to the atypical echographic features of the lesion and the poor response to anticoagulation therapy, a contrast-enhanced computed tomography (CT) scan was performed for further evaluation (Fig. 2). Lesion diameter was measured on axial images, while longitudinal extent was assessed on reconstructed coronal images. The initial CT scan showed a hypodense, intraluminal lesion in the left common femoral vein, measuring 7.4 cm in length. A follow-up CT conducted 1 year later showed interval growth, with an increase in diameter from 2.0 to 2.8 cm and the longitudinal length from 7.4 to 8.0 cm. Additionally, extension into the external iliac vein and deep femoral vein was also noted. These findings raised concerns that the lesion was more likely an intraluminal mass rather than DVT.

**Figure 3.**
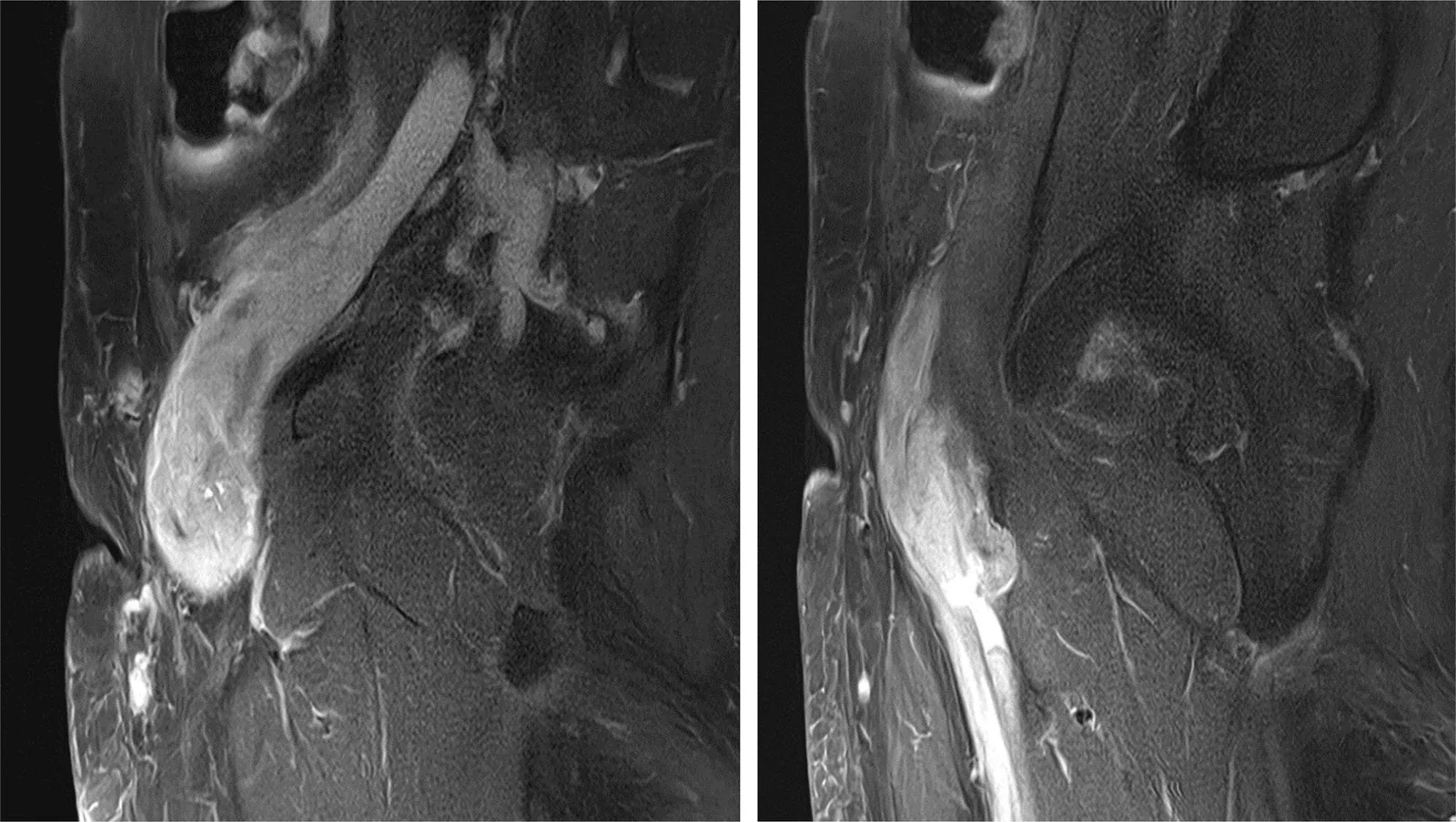
Sagittal view of the magnetic resonance image (MRI) showing an intraluminal mass involving the femoral vein and extending into the external iliac vein. MRI = magnetic resonance image.

**Figure 4.**
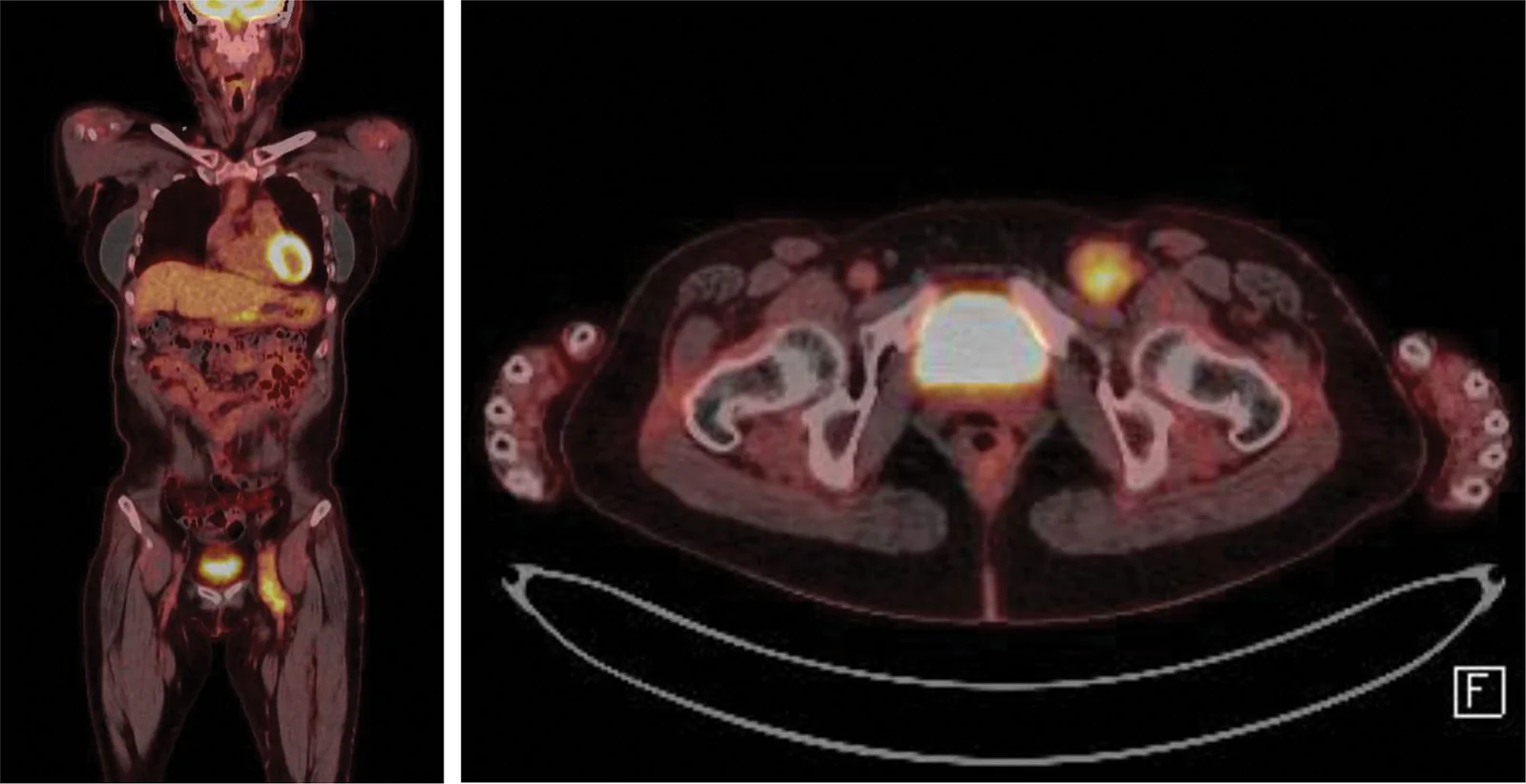
18F-fluorodeoxyglucose (FDG) positron emission tomography-computed tomography (PET-CT) showing a pathologically increased uptake at the left groin level. FDG = fluorodeoxyglucose, PET-CT = positron emission tomography-computed tomography.

To further investigate the nature of the mass, magnetic resonance imaging was performed, which showed a solid mass extending from the external iliac vein to the common femoral vein filling the intraluminal space, indicating a possibility of venous leiomyosarcoma (VLMS) or tumor thrombus. The lesion demonstrated intermediate T1 signal intensity, heterogeneous high T2 signal, and contrast enhancement consistent with a solid intraluminal tumor (Fig. 3). A positron emission tomography-computed tomography scan was done to assess distant metastasis, which showed a focal pathological fluorodeoxyglucose uptake of high intensity in the left inguinal area, but no metastatic lesion (Fig. 4).

Surgical resection of the femoral lesion was performed under general anesthesia. A 10 cm longitudinal incision was made along the left inguinal area. The femoral vein and its branches were mobilized, and the great saphenous vein (GSV) was ligated. The inguinal ligament was divided, and dissection was extended to the distal external iliac vein. The tumor extended from the distal external iliac vein through the common femoral vein, originating from the proximal deep femoral vein. The proximal margin of the mass was palpable, allowing enough space for vein clamping. Prior to clamping, 3000 IU of intravenous unfractionated heparin was administered per institutional protocol for venous reconstruction.

**Figure 5.**
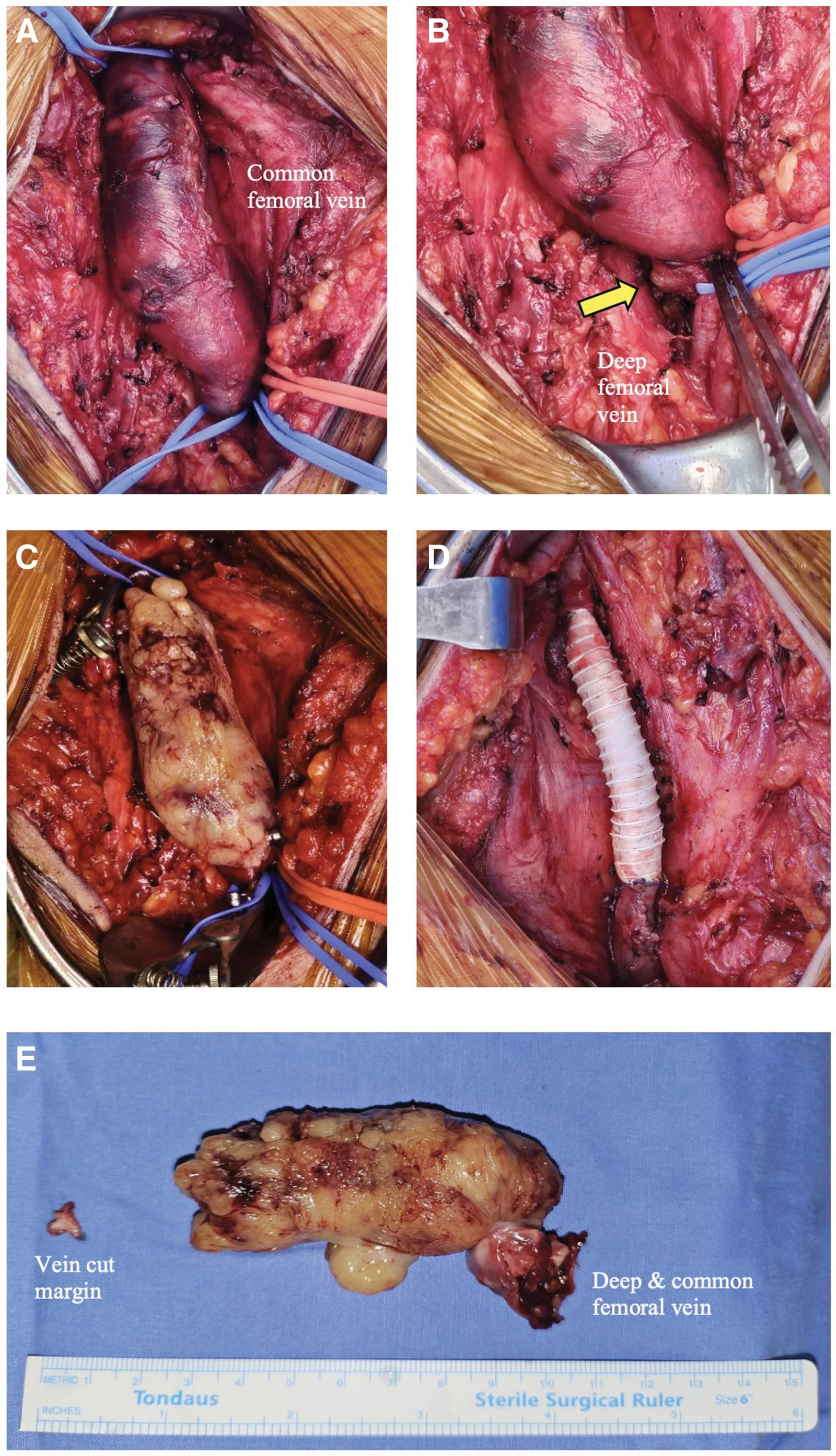
(A) Exposure of the left common femoral vein (CFV) encasing a visible intraluminal mass. (B) Tumor infiltrating a portion of the proximal deep femoral vein (arrow). (C) Exposure of the entire intraluminal mass after venotomy. (D) Graft interposition using a ringed 8 mm expanded polytetrafluoroethylene (ePTFE). (E) Resected mass including the femoral vein, and additional vein cut margin. CFV = common femoral vein, ePTFE = expanded polytetrafluoroethylene.

Upon venotomy, an 8 cm irregular, firm, whitish intraluminal mass was identified, adherent to a small segment of the proximal deep femoral vein wall which was presumed to be the origin of the mass (Fig. 5A, B). En bloc resection was performed, removing the entire mass along with the segment of the femoral vein in contact with the lesion. Graft interposition was done to reconstruct the femoral vein using an 8 mm expanded polytetrafluoroethylene graft (Fig. 5C, D). An enlarged inguinal lymph node was also identified and resected to check for metastatic involvement. The entire mass and the vein resection margins were sent for pathologic examination (Fig. 5E).

Final histopathology revealed an atypical spindle cell proliferative lesion, with a mitotic count score of 26 per 10 high-power fields and focal necrosis. The mitotic activity was consistent with high-grade LMS. Immunohistochemical staining (IHCS) was positive for actin and desmin, and negative for protein S-100 and pancytokeratin – findings consistent with LMS. While the vein resection margin and the resected lymph node were both negative for malignancy, the external surface of the mass showed tumor cell involvement of the external surface without involvement of the transected vein margin.

The patient had an uneventful postoperative recovery and resumed DOAC to prevent DVT and graft occlusion. She was referred to oncology for adjuvant treatment and subsequently received adjuvant chemotherapy including doxorubicin and ifosfamide and radiotherapy targeting the iliac and femoral veins. Follow-up CT performed 3 months after the initial operation showed thrombotic occlusion of the PTFE graft. The patient experienced mild leg edema which resolved with conservative management. Magnetic resonance imaging was performed every 3 months, with no evidence of disease recurrence at 12-month follow-up.

This case report was approved by the Institutional Review Board (IRB) of Seoul St. Mary’s Hospital, the Catholic University of Korea (KC24ZISI0827). The need for informed consent was waived due to the retrospective nature of the study and minimal risk to the patient.

## 3. Discussion

VLMS is an extremely rare subgroup, accounting for <2% of all LMS. It is the most common primary malignancy of the vascular system, with a fivefold higher incidence in veins compared to arteries. Known for its aggressive nature, VLMS has a high metastatic rate of approximately 50% at time of initial diagnosis, most often metastasizing to the lungs and liver. Over half of VLMS cases originate in the inferior vena cava, whereas infrainguinal involvement is far less common, with the saphenous, femoral, and tibial veins being the most commonly origin, in that order.^[4-7]^ Reported survival rates range from 31% to 62%, with a 10-year survival rate as low as 22%.^[8,9]^ Given the poor prognosis, early diagnosis is crucial to avoid delays in treatment.

Duplex ultrasound is commonly employed as the first-line imaging modality for evaluating leg edema. However, VLMS can be misdiagnosed as DVT due to overlapping clinical presentations, making differentiation challenging with ultrasonography alone. In our case, follow-up ultrasonography performed 9 months after the initial exam showed progression of the lesion with increased venous diameter. Given that chronic venous thrombosis typically leads to reduction of vein diameter over time, this can be a clue suggesting possibility of VLMS. Progressive venous diameter enlargement, persistence despite anticoagulation, preserved Doppler flow, and interval growth on CT were key clues arguing against chronic DVT. Also, the presence of color Doppler flow in both the deep and superficial femoral veins, despite a hypoechoic mass occupying the entire femoral vein lumen, was also inconsistent with DVT and more suggestive of VLMS. Previous studies have highlighted the value of a multimodal ultrasonographic approach – including B-mode, color Doppler, pulsed-wave Doppler, and contrast-enhanced ultrasound – in distinguishing VLMS from DVT.^[3,10]^ Zhang et al demonstrated that contrast-enhanced ultrasound can identify rapid, heterogeneous, and intense enhancement during the arterial phase, reflecting the hypervascularity of the mass.^[10]^

There are no specific laboratory or radiologic tests available to definitively diagnose VLMS. The only method available to confirm the diagnosis is histopathologic examination. Tissue samples can be obtained through fine needle aspiration or core needle biopsy, while open incisional or excisional biopsy may also be considered, depending on the size and location of the mass.^[11,12]^ Although preoperative biopsy can assist in planning the surgical approach and may be considered in some institutions, we chose not to perform a preoperative needle biopsy in this case due to the potential risks of bleeding and the possibility of tumor seeding. Given the mass’s relatively superficial location, curative excision was feasible without prior biopsy.

Due to the limited number of reported cases, there is no clinical consensus on the optimal choice of conduit for reconstruction or the long-term graft patency. Reconstruction options after resection of LMSs of the inferior vena cava include ligation without repair, resection followed by primary repair, patch angioplasty, or graft interposition using autologous or prosthetic conduits.^[13,14]^ Spark et al reported 4 cases of wide excision of VLMS followed by reconstruction using GSV, all of which remained patent until the patient’s death.^[15]^ In our case, we used an externally supported expanded polytetrafluoroethylene graft to prevent graft compression, along with a therapeutic dose of DOAC. However, the graft became occluded after 3 months postoperatively. Had the GSV been suitable, using an autologous graft may have offered superior long-term patency.

Currently, there are no randomized trials evaluating the role of adjuvant radiation or chemotherapy in the treatment of VLMS. Existing studies have reported the use of adjuvant radiation therapy in high-grade or malignant cases, while chemotherapy is typically reserved for refractory or metastatic disease.^[1,3,6]^ In our case, adjuvant chemotherapy and radiotherapy were selected due to high-grade histology and concern for microscopic residual disease following external surface involvement. Although data on the specific responsiveness of VLMS to radiation therapy are limited, adjuvant radiotherapy is often employed to reduce the risk of local recurrence and invasion into adjacent soft tissues. Despite the relatively indolent growth pattern of these tumors, up to 50% to 60% of patients with VLMS present with metastatic disease at the time of surgical resection, contributing to the overall poor prognosis. Patients with VLMS arising from the saphenous vein in particular tend to have a worse prognosis, largely due to delayed diagnosis at an advanced stage. Effective management requires close collaboration between surgical and oncology teams.

## 4. Conclusion

In this paper we described a case of a patient diagnosed with a femoral vein LMS which is extremely rare. Initial misdiagnosis highlights the importance of a multimodal approach in cases with atypical clinical courses. Wide excision with negative margins remains essential for curative treatment.

This case highlights characteristic diagnostic delay patterns and reconstructive outcomes in deep femoral vein leiomyosarcoma. Our experience emphasizes the value of a multimodal diagnostic approach in distinguishing neoplasms from thrombosis and underscores the importance of maintaining a high level of suspicion for malignancy, especially when suspected DVT follows an unusual course.

## Author contributions

**Conceptualization:** Eunju Jang.

**Data curation:** Eunju Jang.

**Investigation:** Eunju Jang, Sang Seob Yun.

**Supervision:** Sun Cheol Park, Jang Yong Kim, Sang Seob Yun.

**Validation:** Ki Yoon Moon, Sun Cheol Park, Jang Yong Kim, Sang Seob Yun.

**Writing – original draft:** Eunju Jang.

**Writing – review & editing:** Eunju Jang, Ki Yoon Moon, Sun Cheol Park, Jang Yong Kim, Sang Seob Yun.
